# Identification of Tyrosinase Inhibitors and Their Structure-Activity Relationships via Evolutionary Chemical Binding Similarity and Structure-Based Methods

**DOI:** 10.3390/molecules26030566

**Published:** 2021-01-22

**Authors:** Prasannavenkatesh Durai, Young-Joon Ko, Jin-Chul Kim, Cheol-Ho Pan, Keunwan Park

**Affiliations:** 1 Natural Product Informatics Research Center, KIST Gangneung Institute of Natural Products, Gangneung 25451, Korea; prasanna@kist.re.kr (P.D.); yjko@kist.re.kr (Y.-J.K.); jckim@kist.re.kr (J.-C.K.); panc@kist.re.kr (C.-H.P.); 2 Department of Bioinformatics and Life Science, Soongsil University, Seoul 06978, Korea

**Keywords:** evolutionary chemical binding similarity, ECBS, tyrosinase inhibitors, activity cliffs, pharmacophore, molecular docking

## Abstract

Tyrosinase is an enzyme that plays a crucial role in the melanogenesis of humans and the browning of food products. Thus, tyrosinase inhibitors that are useful to the cosmetic and food industries are required. In this study, we have used evolutionary chemical binding similarity (ECBS) to screen a virtual chemical database for human tyrosinase, which resulted in seven potential tyrosinase inhibitors confirmed through the tyrosinase inhibition assay. The tyrosinase inhibition percentage for three of the new actives was over 90% compared to 61.9% of kojic acid. From the structural analysis through pharmacophore modeling and molecular docking with the human tyrosinase model, the pi–pi interaction of tyrosinase inhibitors with conserved His367 and the polar interactions with Asn364, Glu345, and Glu203 were found to be essential for tyrosinase–ligand interactions. The pharmacophore features and the docking models showed high consistency, revealing the possible essential binding interactions of inhibitors to human tyrosinase. We have also presented the activity cliff analysis that successfully revealed the chemical features related to substantial activity changes found in the new tyrosinase inhibitors. The newly identified inhibitors and their structure–activity relationships presented here will help to identify or design new human tyrosinase inhibitors.

## 1. Introduction

Tyrosinase is an essential enzyme in melanin synthesis, contributing to pigmentation in mammals [1]. Tyrosinase catalyzes the hydroxylation of l-tyrosine to l-DOPA, which is the first step in melanogenesis [1]. Further, l-DOPA is oxidized by tyrosinase to dopaquinone in the melanin synthesis pathway [1]. The tyrosinase-related proteins (TRPs) are also important in melanin synthesis through a few reactions, and their active site contains two Zn^2+^ ions unlike two Cu ions in tyrosinase [1,2]. Tyrosinase also causes browning in food products, which damages the appearance in fruits and vegetables, after it oxidizes the phenolic substrates during various handling processes in the food industry [3,4]. Thus, tyrosinase inhibitors can have industrial applications such as skin-whitening [5] and antibrowning agents [3]. A variety of natural or synthetic tyrosinase inhibitors have been reported, but effectiveness in clinical trials are yet to be confirmed for many of them [6,7]). Thus, researchers continue to adopt several in vitro and in silico procedures to identify clinically effective tyrosinase inhibitors and try to fulfill the industrial demands [6]. In this study, we applied chemical similarity-based virtual screening (VS) to select the candidates for tyrosinase inhibitors by using evolutionary chemical binding similarity (ECBS) [8], a machine learning-based similarity model recently developed by our group.

Screening virtual chemical libraries in the early steps of drug discovery can be a time and cost-saving approach because it requires minimal resources [9,10]. Several ligand-based 2D and 3D chemical similarity approaches are available [11] to screen the vast number of chemicals. Still, many of them focus only on structural similarity from the molecules that bind to a single target, which may miss the important spatial 3D structural features related to biological activity [12]. Similarly, the phenotypic similarity method, which calculates similarity based on comparing functions of chemicals, has limited usage in VS due to a lack of data [13]. To overcome those limitations, we recently developed the evolutionary chemical binding similarity (ECBS) method [8] that defines chemical similarity in terms of their target binding similarity; chemicals of high ECBS scores likely bind to identical target proteins. Among the variants of ECBS models, target-specific ensemble ECBS (TS-ensECBS) successfully identified new inhibitors for Serine/arginine protein kinase 1 (SRPK1) [8], SRPK2 [8], MEK1 [14], and EPHB4 [14].

In the current study, we revealed seven promising tyrosinase inhibitors confirmed through the tyrosinase inhibition assay. Based on the experimental results, the key atomic features of tyrosinase inhibitors and the residues they possibly interact with are presented through pharmacophore modeling and molecular docking with the human tyrosinase model. Finally, activity cliffs (ACs) in the molecules that exhibit drastic changes in tyrosinase inhibition due to minor structural differences are revealed through molecular matching pairs [15]. During statistical validation, the selected pharmacophore model has clearly differentiated the new actives from known inactives of human tyrosinase and decoys. Besides, the molecular docking study agrees with pharmacophore features, ACs, and experimental results from our study, presenting a reliable binding interaction model of human tyrosinase.

## 2. Results

### 2.1. ECBS-Based Virtual Screening and Tyrosinase Inhibition Assay

**Figure 1:**
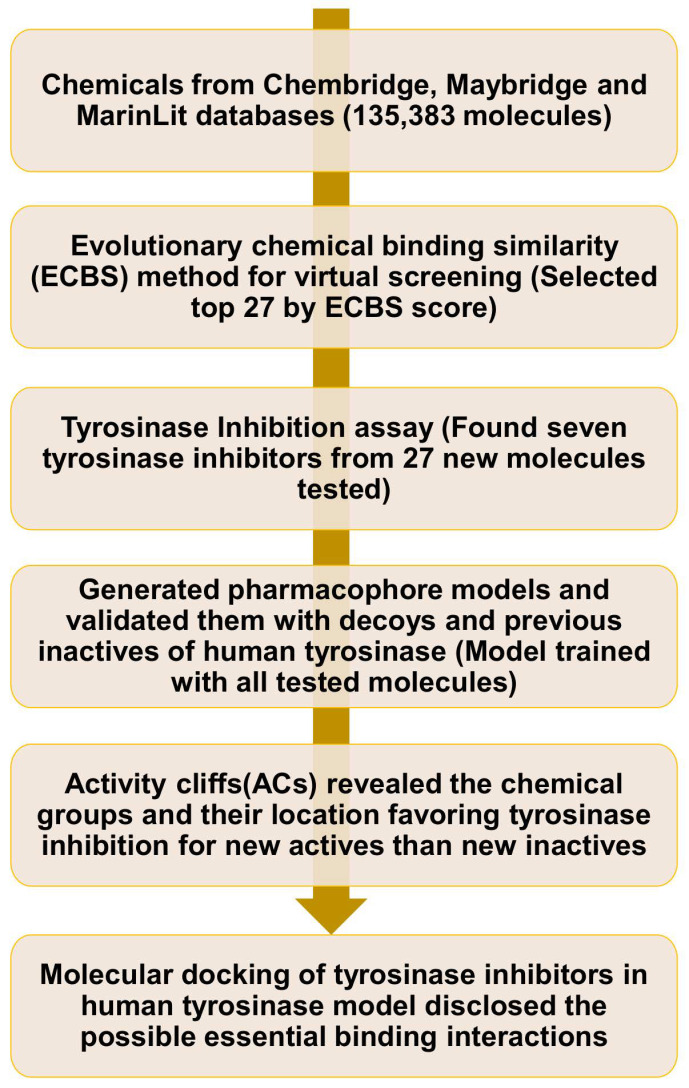
The schematic of the virtual screening procedure followed by pharmacophore modeling, activity cliff analysis, and molecular docking study for tyrosinase inhibitors.

**Figure 2:**
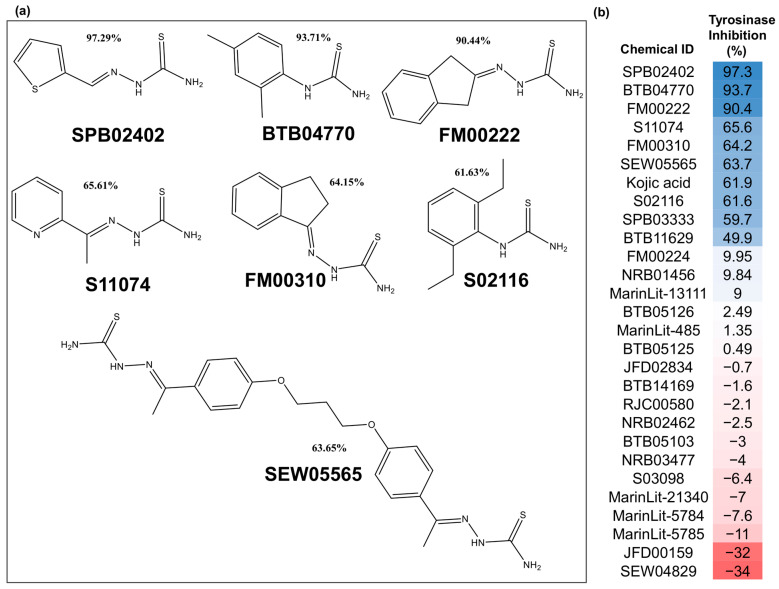
(**a**) Two-dimensional structures of the newly identified tyrosinase inhibitors found in the present study. (**b**) The relative tyrosinase inhibitory activities for 27 chemical compounds (1 mM) tested through the tyrosinase inhibition assay are shown. The tyrosinase inhibition percentage values are represented by a color gradient from blue (highest) to red (lowest).

The flowchart of the entire study is shown in Figure 1. From the VS through TS-ensECBS, the top 27 molecules (by cutoff score 0.74 for natural products and 0.85 for synthetic molecules) were chosen along with kojic acid as a positive control for the tyrosinase inhibition assay (Figure 2 and Figure S1). Out of them, seven new actives exhibited effective inhibition of tyrosinase (Table 1), and three among them had an inhibition percentage of more than 90, which was higher than kojic acid, a well-known tyrosinase inhibitor. The two new actives, SPB02402 and BTB04770, structurally represent all the seven tyrosinase inhibitors found in this study and show the highest tyrosinase inhibition percentage among others (Figure 2).

### 2.2. Pharmacophore Models

#### 2.2.1. Common Feature Pharmacophore Models

The tyrosinase inhibition values from the enzyme assay were used along with 3D structures of chemicals to generate the pharmacophore models. The inhibition percentage values at 1 mM concentration were used for all the chemicals except SEW05565, which had higher inhibition at 0.1 mM than 1 mM concentration. We used the common feature pharmacophore models function in Discovery Studio (DS) 2019 [16] to obtain the pharmacophore features with spatial arrangements responsible for binding to tyrosinase. In the pharmacophore modeling, every input molecule had its activity information labeled only as active or inactive. The chemicals with tyrosinase inhibition values higher than 60% were considered actives, and the remaining 20 molecules were considered inactives. The common feature pharmacophore models from DS only consist of the extracted features from tyrosinase inhibitors without any refinement. The DS accepts a maximum of five features to get in the output model, so we viewed all the ligand features in DS before choosing five. The selected features are the hydrogen bond acceptor (HBA), hydrogen bond donor (HBD), hydrophobic (HY), ring aromatic (RA), and zinc binder (ZB). In total, 10 models were generated, most of which contained similar pharmacophore features with different inter-feature distances. We also got another 10 models without including the ZB feature to check the importance of this atom type.

#### 2.2.2. 3D-QSAR Predictive Pharmacophore Models

We generated 3D-QSAR predictive pharmacophore models using experimentally tested chemicals with their inhibition percentages as activity values. Unlike the common feature pharmacophore models discussed above, the 3D-QSAR pharmacophore model considers the exact activity values (tyrosinase inhibition percentage) of molecules when generating the predictive pharmacophores. These pharmacophore models may not only have exact features present in the input chemicals but also the features after minor perturbations by the Catalyst Hypogen algorithm implemented in DS [17]. A statistical validation score of every model is given as an output. We allowed the input activity data to be adjusted up to 4 orders of magnitude. In total, 20 3D-QSAR models were obtained, and half of them were not allowed to have ZB as one of the features.

#### 2.2.3. Validation and Selection of Pharmacophore Models

We prepared the following four chemical sets to validate the 40 pharmacophore models generated through the common feature pharmacophore models and the 3D-QSAR predictive pharmacophore methods; (1) new actives—the seven chemicals proved in the tyrosinase inhibition assay as high-affinity binders and their inhibition percentage was higher than 60%, (2) new inactives—the 20 molecules that were either moderate or low-affinity binders to tyrosinase with the inhibition percentage of less than 60% in the tyrosinase inhibition assay, (3) previous actives—the 46 high-affinity inhibitors of human tyrosinase (IC_50_ values less than 10,000 nM) from BindingDB database, and (4) previous inactives—the 56 moderate- or low-affinity binders of human tyrosinase (IC_50_ values higher than 10,000 nM) from BindingDB database [18]. In addition, the 400 chemical decoy set similar to the new actives was generated from the DUD-E database to validate the models [19]. The chemical duplicates were removed in DS before further calculations.

The fit values from all the 40 models of the previous inactives tested with human tyrosinase (Table S1) and decoys were further used along with the scores of new actives to validate and choose the best model. The chemicals that map best to the pharmacophore model will have a fit value of 1, and the ones that do not fit at all to the features will have 0. Among the test set chemicals, 12 molecules from BindingDB (six previous actives and six previous inactives) and 16 decoys did not output any fit score for any of the input pharmacophore models when mapped by the Ligand Profiler in DS.

**Figure 3:**
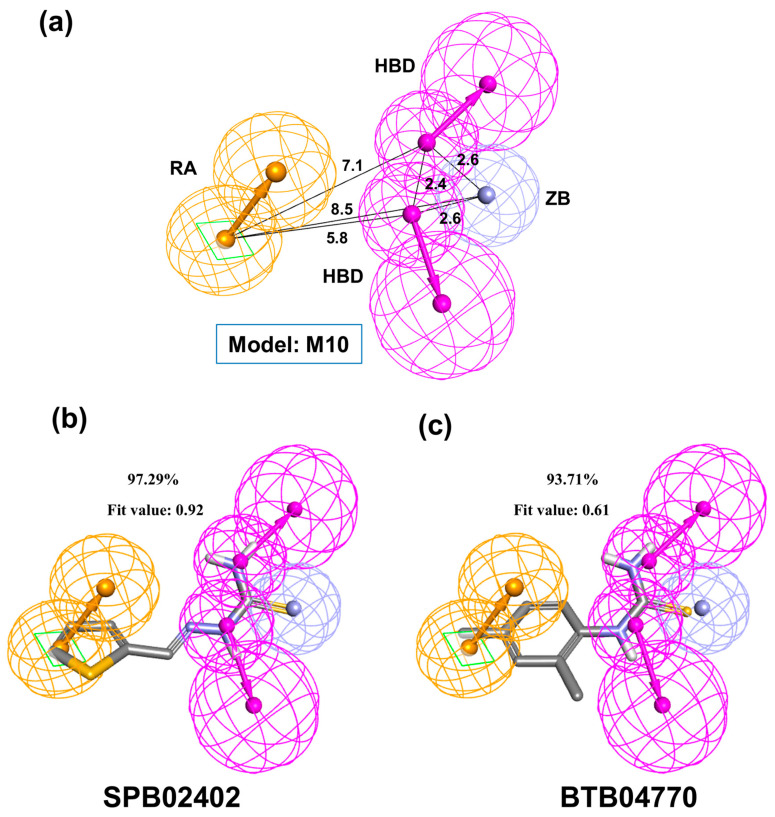
The tyrosinase pharmacophore model M10 mapped to the two representative inhibitors. (**a**) The common feature pharmacophore model M10 generated using chemicals tested in our study. The interfeature distances are given in angstroms. Hydrogen bond donor (HBD), ring aromatic (RA), and zinc binder (ZB) are the features extracted from chemicals. (**b**) SPB02402 and (**c**) BTB04770 mapped to the pharmacophore model M10. Their tyrosinase inhibition percentages and pharmacophore fit values are given.

For all the 40 pharmacophore models, we calculated the area under the curve (AUC) score in the precision–recall (PR) curve to choose the best model based on how well they classify the new actives from previous inactives, decoys, and new inactives (Table S2). Among the 40 pharmacophore models, features in model M10 (obtained through the common feature pharmacophore models) (Figure 3) look promising because its classification performance was more consistent than the others (Table S2). The PR AUC value of M10 was 0.92 (SPB03333 with 59.7% inhibition was considered active) when it classified the new actives from new inactives, previous inactives, and decoys together (Table S2 and Figure S2). During the individual classification of validation sets from new actives, the PR AUC values of M10 were 1, 0.93, and 0.95 for previous inactives, decoys, and new inactives, respectively (Table S2 and Figure S2). Additionally, the receiver operating characteristic (ROC) curve showed the consistent results that the ROC AUC values of M10 were 1, 0.99, and 0.97 for previous inactives, decoys, and new inactives, respectively (Figure S3). The mappings of all the new actives to M10 are shown along with their pharmacophore fit scores (Figure 3 and Figure S4).

### 2.3. Homology Modeling and Molecular Docking

We chose the TRP1-3M mutant protein in complex with kojic acid (PDB id: 5M8Q) as the modeling template. The TRP1-3M mutant structure was chosen because the three residues mutated in the active site are corresponding to the ones in human tyrosinase [2]. We got 20 optimized human tyrosinase models from DS and used the MODELLER DOPE score [20] to choose the best model among the protein conformations. The protein model was validated with a few rotamer libraries and Ramachandran map in DS. No incorrect main-chain conformations or side-chain deviations were found in the active site residues, and 96.5% of residues were in the allowed region. Additionally, the compatibility score from the Verify3D server [21] for 97.03% of residues in the model was higher than 0.2, representing high model reliability.

**Figure 4:**
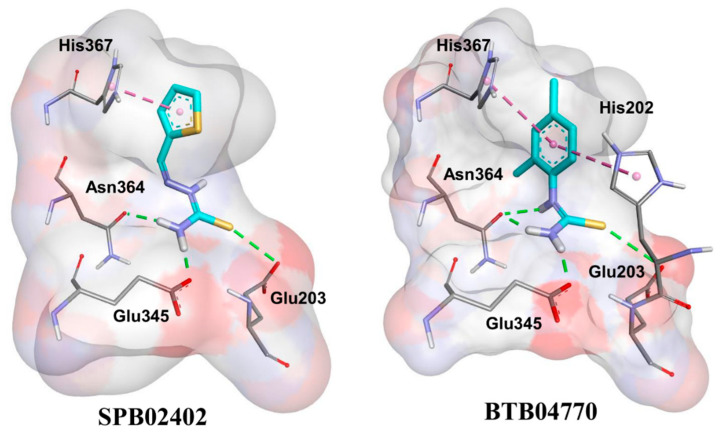
Molecular docking performed for the two representative inhibitors in the human tyrosinase model structure. The tyrosinase inhibitors and tyrosinase amino acids are shown as cyan and grey sticks, respectively. The pi–pi interactions and hydrogen bonds are shown as dotted lines. Only the interacting residues are shown for clarity by hiding the Cu ions, water molecule, and other tyrosinase residues in the surface background of human tyrosinase’s active site.

From the docking results of 1000 poses, we sorted the lowest energy conformation in the cluster with a high number of poses. We obtained the docking poses for two tyrosinase inhibitors that structurally represent all the new actives (Figure 4). As expected, pi–pi interaction with His367 was conserved among the inhibitors. In addition, the polar interactions with Asn364, Glu345, and Glu203 were conserved. For BTB04770 and similar structures, His202 might play a key role in tyrosinase inhibition. The autodock binding energy for the BTB04770 and SPB02402 were −5.06 and −4.78 kcal/mol, respectively.

### 2.4. Activity Cliffs

**Figure 5:**
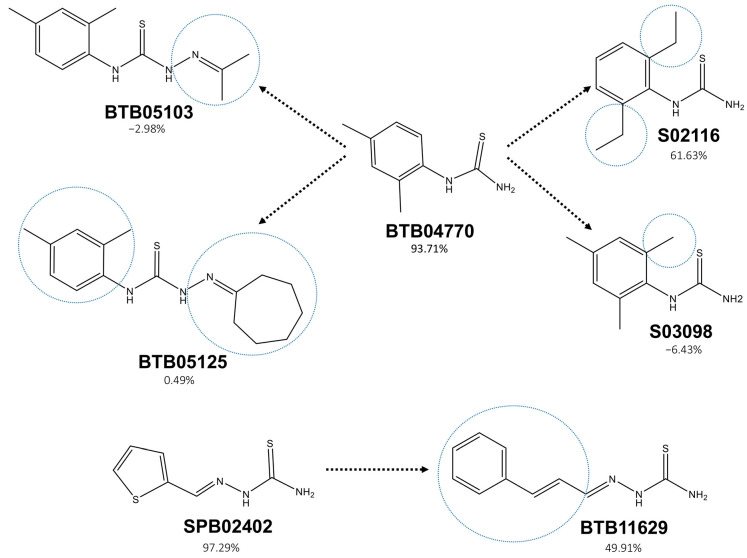
Activity cliffs of the molecules tested in the tyrosinase inhibition assay are presented. The tyrosinase inhibition activities of the two representative chemicals (BTB04770 and SPB02402) are compared with the other chemicals in terms of their activity changes. The blue dotted circles exhibit the structural difference(s) between the molecules. The tyrosinase inhibition percentage of every molecule is given.

The new actives and new inactives were compared with pairwise structural similarity and notable opposite activity. The new molecules were used in DS along with their tyrosinase inhibition percentage values to get the ACs using matched molecular pairs information. There were crucial differences in the activity values between the structurally similar BTB04770 and BTB05103; BTB04770 and S03098; BTB04770 and BTB05125; BTB04770 and S02116; and SPB02402 and BTB11629 (Figure 5). Thiourea was conserved among the inhibitors BTB04770, S03098, and S02116, but there were huge differences among their tyrosinase inhibition properties. The extra methyl group in the 6th position of the phenyl group in S03098 compared to BTB04770 made a huge impact on tyrosinase activity (Figure 5). As understandable from Figure 4, the imidazole ring of His202 might interfere in the key interactions of S03098 by avoiding its stretching due to the hindrance from this extra methyl group and make S03098 inactive. The same reason may also have influenced the reduced tyrosinase inhibitory activity of S02116 due to its ethyl group in the 6th position of the phenyl group (Figure 5). In the case of BTB05103 and BTB11629, any of the predicted key polar interactions by their amino group (Figure 4) with Asn364 and Glu345 may be missing because of their lengthy carbon chains. The tyrosinase activity differences among the molecules and the minor differences in their chemical groups are shown in Figure 5.

## 3. Discussion

In this study, seven tyrosinase inhibitors were newly identified by testing 27 chemicals (success ratio 25.9%) through the TS-ensECBS method (Table 1 and Figure 2). The moderately active inhibitors made the success percentage increase to 33.3%. The tyrosinase inhibition activity for seven molecules was even higher or similar to kojic acid, and three among them had over 90% tyrosinase inhibition (Figure 2b). The two tyrosinase inhibitors, SPB02402 and BTB04770, contained the two conserved structures found among all the new actives and served as representative structures (Figure 2a). The molecules BTB04770 and S02116 had thiourea scaffold in common, and the other five inhibitors, including SPB02402, had the same hydrazinecarbothioamide group. The pharmacophore model M10 generated based on the tyrosinase inhibitory assay results showed consistent classification performance for the test chemical sets (Figure S2). It suggested that the M10 model represents the critical pharmacophore features required for tyrosinase inhibition, which can be used for further tyrosinase inhibitor studies.

The ACs data in Figure 5 gives additional information on active and inactive groups present in the tested chemicals, which helps focus on the specific chemical group or position to develop new tyrosinase inhibitors. In the analysis of molecular matched pairs, even a single chemical group drastically altered the tyrosinase inhibitory activity (Figure 5). The existence of ACs suggests that predicting the tyrosinase inhibition activity only from a few common structural features can be hard. Thus, the newly identified inhibitors in the present study will provide useful information on the structure–activity relationship for tyrosinase.

When it comes to the binding model, the conserved His367 residue interaction can be seen in tyrosinase inhibitors as found in the crystal structures of both TRPs and tyrosinase of *Agaricus bisporus* [2,22]. Similarly, His202 seems to be influencing the tyrosinase inhibition of molecules based on the chemical group present in the 6th position of the ligand’s phenyl group (Figure 4 and Figure 5). Interestingly, conserved interactions with few residues by the new inhibitors are observed, and they provide valuable insights for the possible binding mode of inhibitors in human tyrosinase. The multiple sequence alignment of tyrosinase sequences from human and other species related to food products has shown that several residues in their active sites were identical (Figure S5). Additionally, molecular docking in the mushroom tyrosinase structure (chosen as a representative for species belong to food products) showed that Glu256, Asn260, and His263 had interactions with both the representative inhibitors SPB02402 and BTB04770 (Figure S6). These three residues corresponded to Glu345, Asn364, and His367 in human tyrosinase that also exhibited interactions with the inhibitors in molecular docking (Figure 4). Hence, similar recognition of representative inhibitors by both the human and mushroom species was assumed. Overall, the results from our study could be helpful in further identification or optimization of tyrosinase inhibitors that are commercially required in cosmetics and food industries.

The present study was focused on validation and interpretation of ECBS screening results with the molecular modeling study (pharmacophore, activity cliffs, and molecular docking) rather than experimental validation. Although the tyrosinase inhibition assay at one chemical concentration (1 mM) did not provide potency information, the activity values provided enough information to classify the compounds into active or inactive and to build a meaningful pharmacophore model. Indeed, the finally selected common feature pharmacophore model (M10) was compatible with the low-resolution activity representation (binary compound classification) and showed a reasonable performance. The follow up in vitro functional studies, including the melanin synthesis assay, will be an interesting further work to validate the biological activity of the new tyrosinase inhibitors and build more accurate pharmacophore models.

## 4. Materials and Methods

### 4.1. ECBS-Based Virtual Screening

Around 25,000 marine natural compounds from MarinLit [23] and 113,334 synthetic chemicals from Chembridge and Maybridge databases were used for VS. The TS-ensECBS screening was performed for human tyrosinase as explained in our previous study [14]. TS-ensECBS calculated the chemical binding similarity to the known tyrosinase inhibitors (top 50 high-affinity molecules by IC_50_ from BindingDB) for the screening chemical dataset. The maximum score among the similarity scores to the 50 known inhibitors was used as a final prediction score for each chemical candidate [8]. As a result, the top-scoring 27 chemicals excluding S14458, a known tyrosinase inhibitor, were chosen for the tyrosinase inhibition assay.

### 4.2. Tyrosinase Inhibition Assay

l-tyrosine was used as a substrate to determine tyrosinase inhibition activity. The reaction mixture contained 0.1 M sodium phosphate buffer, 1.5 mM l-tyrosine, and 1500 U/mL mushroom tyrosinase with sample (*R*) and without sample (*R*′). The control mixture contained 0.1 M sodium phosphate buffer and 1.5 mM l-tyrosine with sample (*C*) and without sample (*C*′). Mixtures were incubated for 15 min at 37 °C, then put on ice to block the reaction. The chemical compound samples were prepared with 1 mM concentration. The absorbance (*Abs*) was measured at 490 nm using a microplate reader. The tyrosinase inhibition activity was calculated as below.

(1) $$\mathrm{Tyrosinase}\;\mathrm{inhibition}\;\mathrm{activity}\left(\%\right)\;=\;100\;-\frac{\mathrm{Abs}\;R-\mathrm{Abs}\;C}{\mathrm{Abs}\;R^{\prime }-\mathrm{Abs}\;C^{\prime }}\;\times \;100$$

### 4.3. Pharmacophore Model Generation

The common pharmacophore features in all the new actives were extracted using Hiphop algorithm in the module *Common Feature Pharmacophore Generation* implemented in DS. The ligands were standardized with preprocessing, and 255 maximum conformers were generated within the relative energy of 10 kcal/mol. After using the *feature mapping* option in DS to find out all the available pharmacophore features, the features HBA, HBD, HY, RA, and ZB were given to obtain the top 10 models with the interfeature distance of 1.5. Only one feature was allowed to miss for the input molecules in the output model. An additional molecular property *Principal* was added for the tested chemicals to input their tyrosinase activity. We did input the new actives chemical set with high tyrosinase inhibition percentages as active molecules. The other 20 molecules, which are either moderately active or inactive in tyrosinase inhibition, were given as inactive chemicals. The 3D-QSAR pharmacophore models were generated with the Catalyst Hypogen algorithm [17] in DS as previously described [14]. The tyrosinase inhibition percentages of chemicals were used as their activity values.

The *prepare ligands* function in DS was used to remove the duplicate chemicals. The *Ligand profiler* module in DS was used to map the molecules against pharmacophore models with *flexible* fitting after a maximum of 255 conformers were generated. The PR AUC values were calculated to choose the best model based on its classification performance.

### 4.4. Homology Modeling and Molecular Docking

In DS, we used *Build homology model* and *Verify Protein* (Modeler) protocols to predict and choose the best protein model among others. The sequence of human tyrosinase from Uniprot database (id: P14679) was downloaded and aligned with the sequence in the crystal structure of the human TRP1-3M mutant (PDB id: 5M8Q). The same structure with structure alignment was given as a template to get the top 20 models with the high *optimization level* option. The mushroom tyrosinase structure (PDB id: 2Y9X) was the other receptor used for molecular docking.

The ligand conformers for molecular docking were generated in DS with the parameters of 1000 *best* maximum conformations, energy threshold of 20 (kcal/mol), 0.2 RMSD cut off, and CHARMm minimization forcefield [24]. AutoDock4.2 [25] was used for molecular docking with the chosen homology model. The python and C Shell scripts provided in the AutoDock suite were used to prepare the ligands, receptor, and Autogrid parameters for calculating atomic affinity maps and docking calculations of each ligand. The grid was set with a dimension of 40 Å and center values of −15.715, −5.183, and −25.641 that covers the active site of the TRP1-3M mutant where kojic acid binds. The two Cu ions (in place of Zn^2+^ ions in TRP-3M) and a water molecule (one that kojic acid interacts with) in the TRP1-3M were grafted to the homology model. The “AD4_parameters.dat” file in AutoDock was edited manually to include Cu ion parameters to perform docking. The results were analyzed with “summarize_results4.py” script.

### 4.5. Activity Cliffs

The *ACs* option in DS was used where both new actives and new inactives were given as input, and their tyrosinase inhibition percentages were their activity values. Activity threshold change was given as −99, and maximum 300 ACs were set to display.

## Figures and Tables

**Table 1:** The output values from TS-ensECBS, pharmacophore model, and tyrosinase inhibition assay for the seven newly identified inhibitors are given.

Molecule Name	TS-ensECBS Score	Pharmacophore Fit Value	Tyrosinase Inhibition Percentage (%)
SPB02402	0.91	0.92	97.29
BTB04770	0.92	0.61	93.71
FM00222	0.85	0.84	90.44
S11074	0.91	0.93	65.61
FM00310	0.90	0.90	64.15
SEW05565	0.90	0.99	63.65
S02116	0.89	0.51	61.63

## Data Availability

The data presented in this study are available on request from the corresponding author.

## Supplementary Materials

The following are available online, Figure S1: The two-dimensional structures of the molecules other than the top seven new actives tested in our study, Figure S2: The PR curves of the pharmacophore model M10 for its multiple validation sets, Figure S3: The ROC curves of the pharmacophore model M10 for its multiple validation sets, Figure S4: The tyrosinase inhibitors other than the two representative chemicals are mapped in the chosen pharmacophore model M10, Figure S5: Alignment of tyrosinase sequences from human and other species related to food products, Figure S6: Molecular docking of two representative inhibitors in mushroom tyrosinase crystal structure (PDB id: 2Y9X), Table S1: The pharmacophore fit values of the previous inactives of human tyrosinase, Table S2: The PR AUC values of the 40 pharmacophore models.
