# Supplementary material for: Identification of Tyrosinase Inhibitors and Their Structure-Activity Relationships via Evolutionary Chemical Binding Similarity and Structure-Based Methods
Source: Molecules. 2021 Jan 22;26(3):566. doi: 10.3390/molecules26030566 (PMC7865271; doi:10.3390/molecules26030566)
Supplement: Supplementary file 1 [file molecules-26-00566-s001.pdf]

Supplementary Materials

Identification of tyrosinase inhibitors and their structure-activity relationships via evolutionary chemical binding similarity and structure-based methods

Prasannavenkatesh Durai [1], Young-Joon Ko [1, 2], Jin-Chul Kim [1], Cheol-Ho Pan [1], and Keunwan Park [1,*]

[1] Natural Product Informatics Research Center, KIST Gangneung Institute of Natural Products, Gangneung, 25451, Republic of Korea; prasanna@kist.re.kr, yjko@kist.re.kr, jckim@kist.re.kr, panc@kist.re.kr, keunwan@kist.re.kr
[2] Department of Bioinformatics and Life Science, Soongsil University, Seoul, 06978, Republic of Korea

* Correspondence: keunwan@kist.re.kr; Tel.: +82 33 650 3663

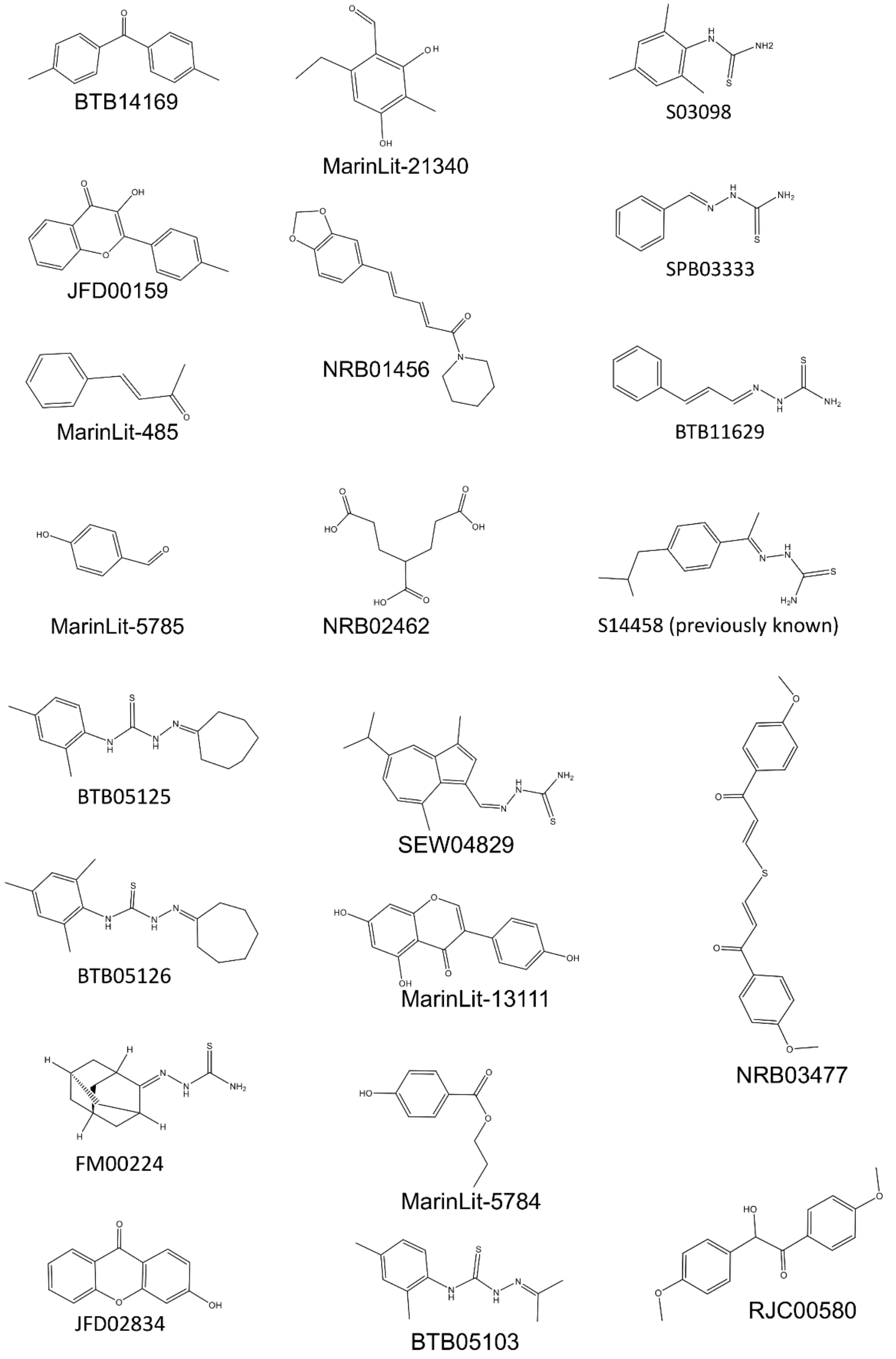

Figure S1. The two-dimensional structures of the molecules other than the top seven tyrosinase inhibitors tested in our study.

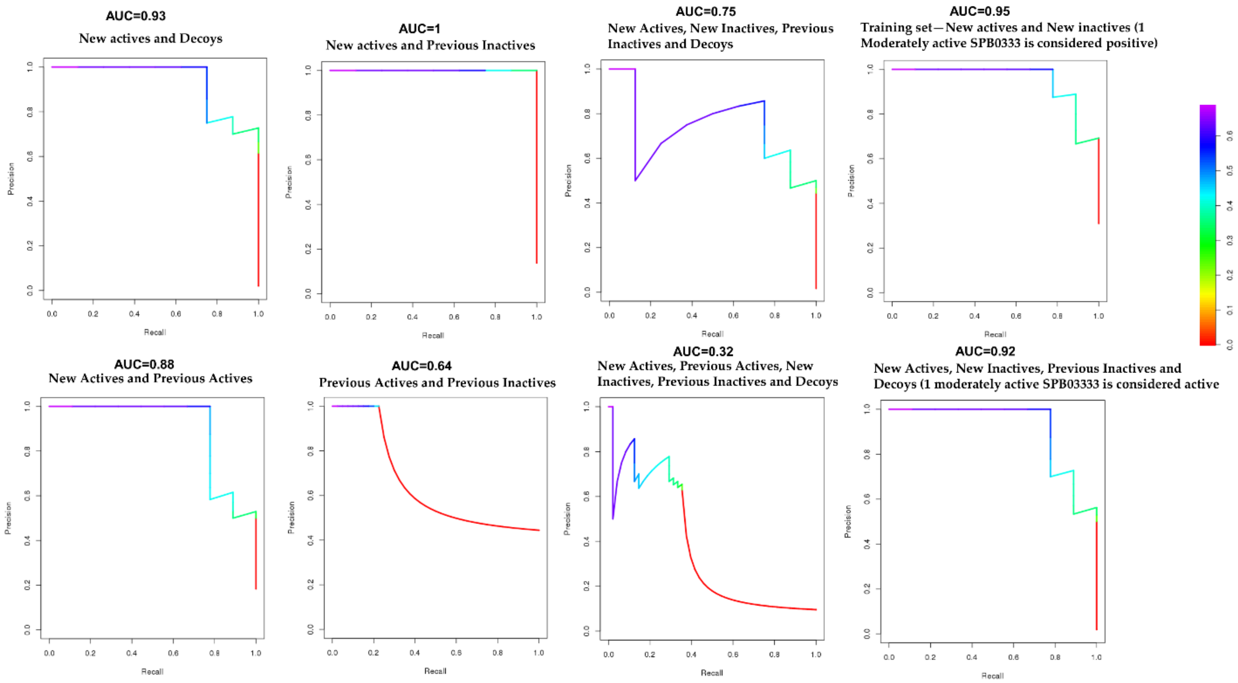

Figure S2. The precision-recall (PR) curves of the pharmacophore model M10 for its multiple validation sets.

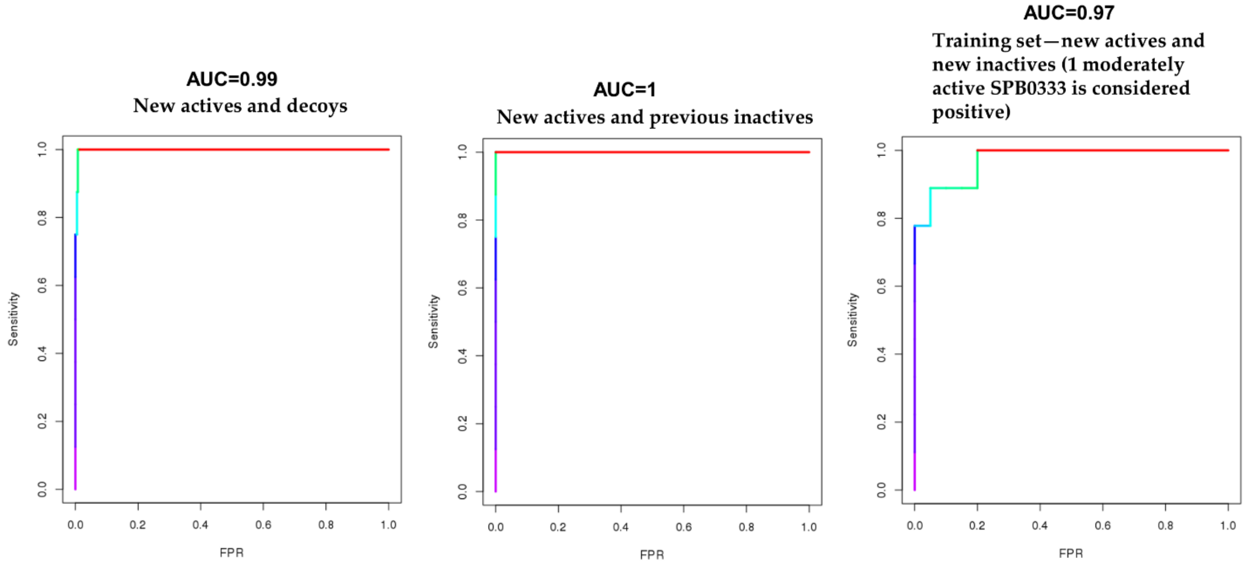

Figure S3. The receiver operating characteristic (ROC) curves of the pharmacophore model M10 for its multiple validation sets

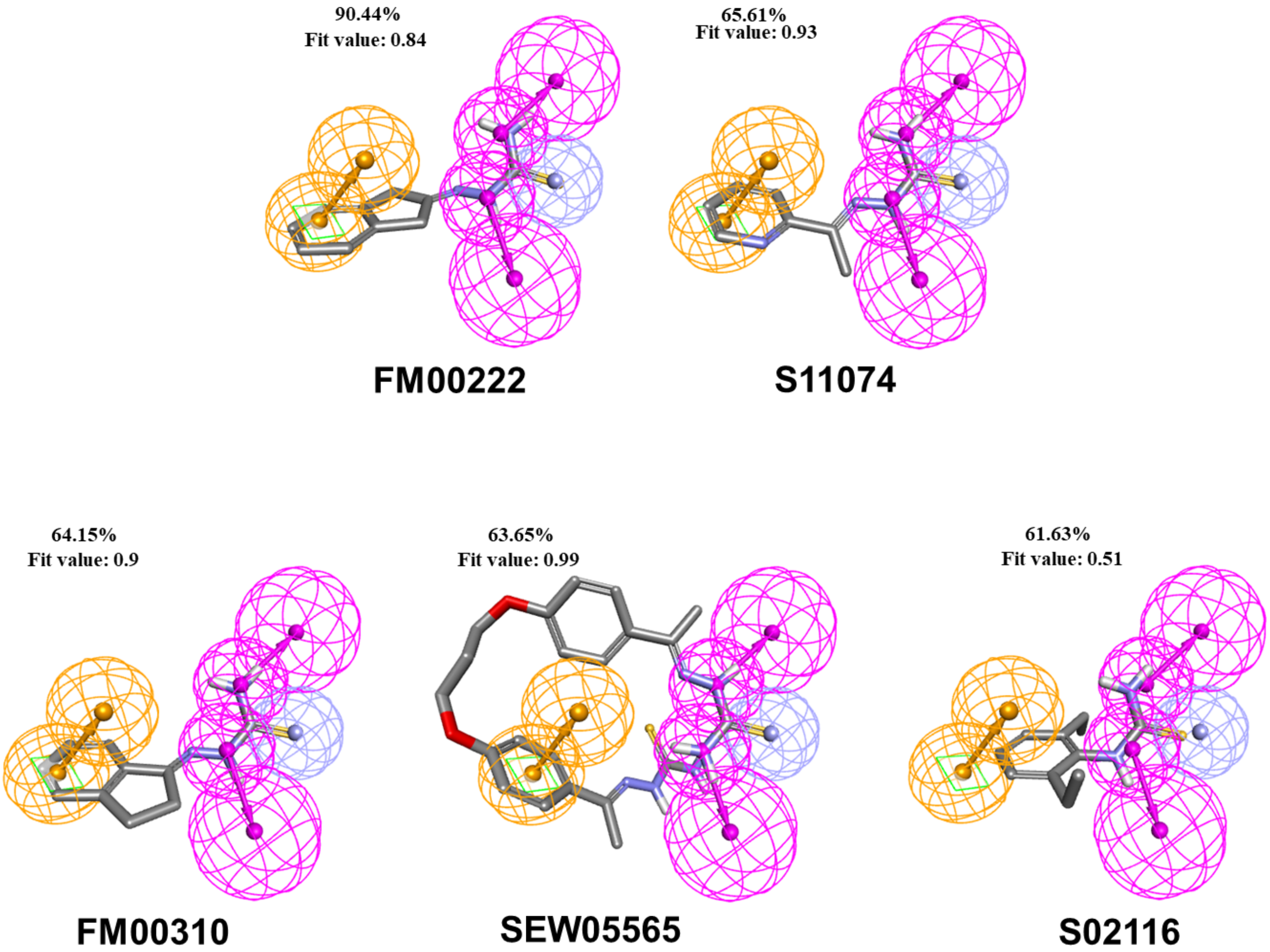

Figure S4. The new actives other than the two representative chemicals are mapped in the chosen pharmacophore model M10.

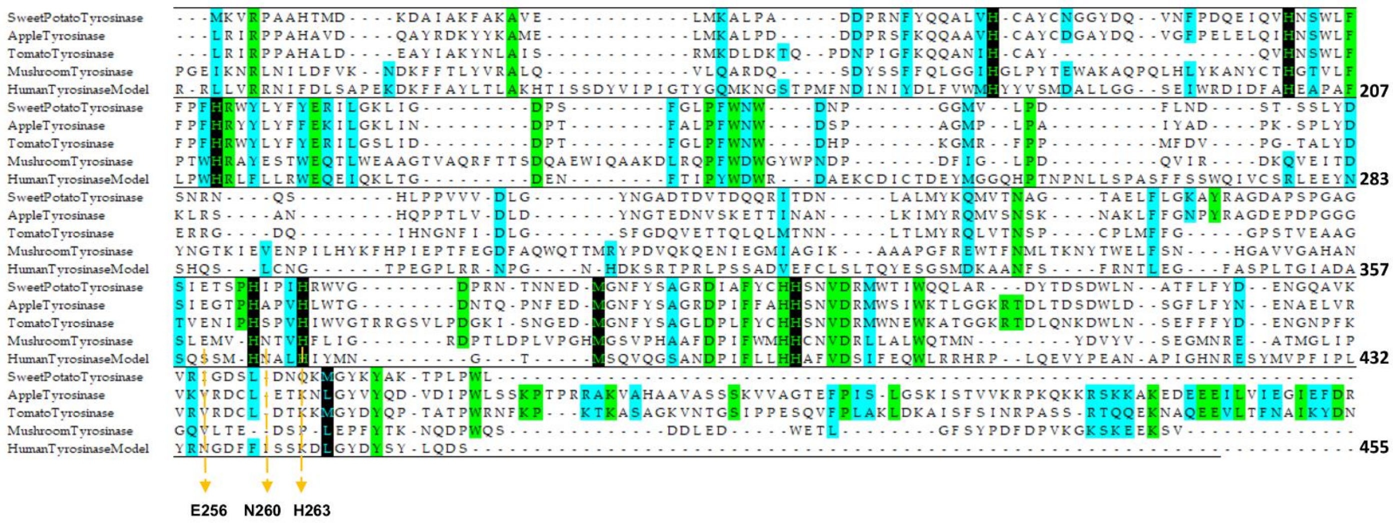

Figure S5. The tyrosinase sequences related to food products in the PDB ids 1BUG (Sweet potato), 2Y9X (Mushroom), 6ELS (Apple), and 6HQI (Tomato) were aligned with the sequence of human tyrosinase. The conserved residues in their active sites are highlighted in black. Sequence numbers of human tyrosinase are labeled at the right end of the sequence. Green color represents the identical residues that are not located in the active site, and cyan represents the residues that are not in the active site but conserved in many species. The three residues (Glu256, Asn260, and His263) indicated by orange dotted lines are from mushroom tyrosinase that shows interactions with inhibitors in molecular docking (Figure S6). These three residues correspond to Glu345, Asn364, and His367 in human tyrosinase that also exhibits interactions with the inhibitors in molecular docking (Figure 4).

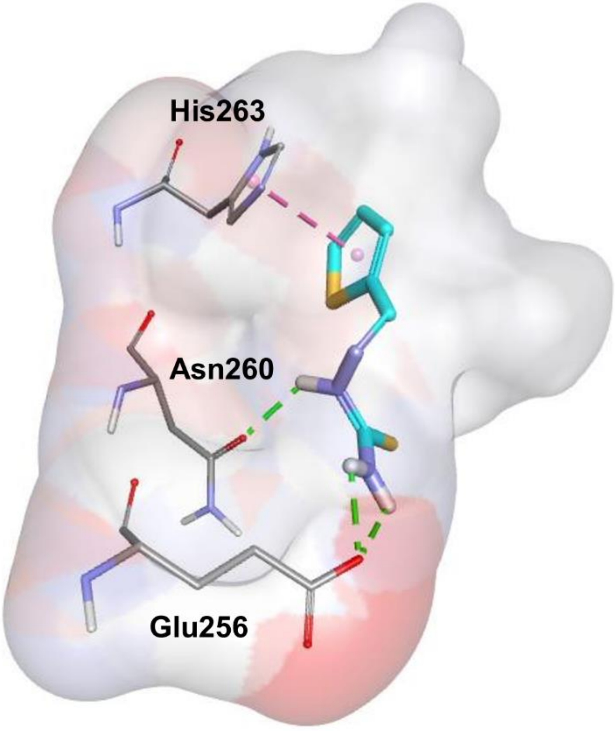

SPB02402

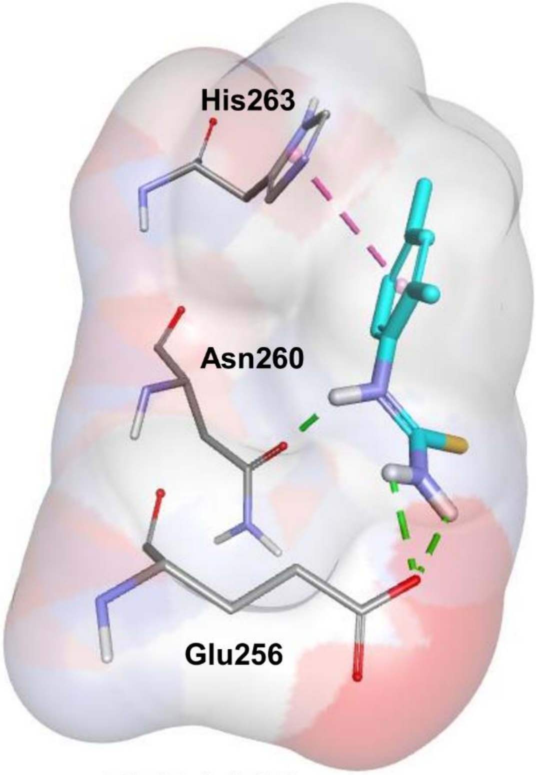

BTB04770

Figure S6. Molecular docking performed for the two representative inhibitors in the mushroom tyrosinase crystal structure (PDB id: 2Y9X). The tyrosinase inhibitors and tyrosinase amino acids are shown as cyan and grey sticks, respectively. The pi-pi interactions and hydrogen bonds are shown as dotted lines. Only the interacting residues are shown for clarity by hiding the Cu ions, and other tyrosinase residues in the surface background of mushroom tyrosinase's active site.

Table S1. The pharmacophore fit values for all the previous inactives of human tyrosinase given in the table were zero.

Chemical ID	BindingDB MonomerID
Molecule	50218206
Molecule1	50218205
Molecule4	50218210
Molecule6	85774
Molecule7	50031467
Molecule9	50242238
Molecule10	50139366
Molecule11	50139367
Molecule12	50139368
Molecule14	50139370
Molecule15	60953
Molecule24	50065387
Molecule25	50351096
Molecule26	50180259
Molecule27	50180261
Molecule28	50193668
Molecule29	50193669
Molecule30	50193670
Molecule32	50108046
Molecule33	50193673
Molecule34	50193672
Molecule35	50219502
Molecule36	50269344
Molecule37	50269342

Molecule38	50269341
Molecule53	50067044
Molecule54	50266759
Molecule55	50067028
Molecule56	50296394
Molecule57	50264832
Molecule58	50296395
resveratrol	23926
Molecule60	50174558
Molecule61	50176525
Molecule62	50096003
Molecule68	50366429
Molecule69	50287120
Molecule70	50287121
Molecule76	50287128
Molecule77	60928
Molecule78	50176696
Molecule79	50370694
Molecule80	50176698
Molecule81	50176699
Molecule82	50176700
Molecule83	50176701
Molecule84	50205806
Molecule85	50205807
Molecule86	50205814
Molecule87	50205815

Table S1 continued…

Table S2. The PR AUC values of the 40 pharmacophore models are given.

Table S2 continued…

**Model**	**New actives, previous actives, new inactives, previous inactives and decoys**	**New actives, new inactives, previous inactives and decoys**	**New actives and decoys**	**New actives and previous inactives**	**Previous actives and previous inactives**	**New actives and previous actives**	**Training set—new actives and new inactives (1 moderately active SPB0333 is considered positive)**	**New actives, new inactives, previous inactives and decoys (1 moderately active SPB03333 is considered positive)**
M1	0.39	0.13	0.15	0.56	0.72	0.26	0.95	0.10
M2	0.43	0.10	0.12	0.42	0.76	0.15	0.86	0.11
M3	0.33	0.60	0.65	0.97	0.58	0.55	0.97	0.65
M4	0.36	0.49	0.57	1	0.58	0.43	0.93	0.55
M5	0.43	0.39	0.43	0.86	0.75	0.37	0.95	0.44
M6	0.37	0.10	0.13	0.42	0.69	0.17	0.91	0.12
M7	0.32	0.68	0.78	0.97	0.57	0.79	0.95	0.80
M8	0.37	0.48	0.54	1	0.58	0.43	0.92	0.52
M9	0.25	0.07	0.08	0.30	0.56	0.25	0.88	0.07
M10	0.32	0.75 (.92 if one moderately active molecule SPB03333 is considered active)	0.93	1	0.64	0.88 (.9 if 1 moderately active SPB03333 is included)	0.95	0.92
M11	0.35	0.75	0.84	0.97	0.58	0.76	0.95	0.84
M12	0.43	0.52	0.66	0.69	0.72	0.59	0.88	0.64
M13	0.41	0.35	0.40	0.74	0.70	0.38	0.94	0.39
M14	0.35	0.11	0.13	0.42	0.72	0.19	0.86	0.12
M15	0.36	0.74	0.79	0.95	0.57	0.55	0.97	0.79
M16	0.40	0.14	0.16	0.53	0.72	0.26	0.95	0.15
M17	0.38	0.20	0.21	0.54	0.70	0.27	0.92	0.20
M18	0.45	0.12	0.14	0.51	0.80	0.15	0.87	0.14
M19	0.40	0.15	0.19	0.45	0.72	0.19	0.91	0.17
M20	0.42	0.20	0.22	0.53	0.70	0.26	0.92	0.20

M21	0.37	0.20	0.21	0.53	0.71	0.26	0.91	0.19
M22	0.48	0.49	0.59	0.80	0.81	0.61	0.87	0.64
M23	0.43	0.52	0.66	0.69	0.73	0.59	0.90	0.64
M24	0.27	0.46	0.54	1	0.55	0.44	0.80	0.48
M25	0.30	0.23	0.28	0.40	0.57	0.34	0.70	0.22
M26	0.22	0.05	0.06	0.38	0.75	0.15	0.70	0.06
M27	0.44	0.14	0.18	0.41	0.74	0.18	0.90	0.16
M28	0.32	0.76	0.85	1	0.58	0.86	0.93	0.85
M29	0.43	0.19	0.20	0.55	0.79	0.26	0.95	0.18
M30	0.39	0.10	0.12	0.40	0.77	0.15	0.91	0.12
M31	0.31	0.63	0.69	0.70	0.56	0.72	0.81	0.72
M32	0.28	0.45	0.52	0.97	0.58	0.50	0.91	0.50
M33	0.41	0.19	0.20	0.57	0.79	0.27	0.97	0.19
M34	0.40	0.02	0.03	0.12	0.62	0.11	0.56	0.03
M35	0.42	0.47	0.52	0.75	0.73	0.48	0.88	0.58
M36	0.36	0.08	0.09	0.41	0.66	0.14	0.98	0.09
M37	0.29	0.04	0.06	0.23	0.57	0.24	0.88	0.05
M38	0.36	0.72	0.89	0.97	0.58	0.79	0.94	0.88
M39	0.42	0.24	0.25	0.72	0.76	0.26	0.90	0.26
M40	0.39	0.39	0.43	0.86	0.71	0.44	0.96	0.44
